# Atypical Anorexia in Youth: Cautiously Bridging the Treatment Gap

**DOI:** 10.3390/children9060837

**Published:** 2022-06-05

**Authors:** Melissa Freizinger, Michelle Recto, Grace Jhe, Jessica Lin

**Affiliations:** 1 Division of Adolescent and Young Adult Medicine, Boston Children’s Hospital, Boston, MA 02115, USA; grace.jhe@childrens.harvard.edu (G.J.); jessica.lin@cchmc.org (J.L.); 2 Department of Psychiatry, Harvard Medical School, Boston, MA 02115, USA; 3 Cincinnati Children’s Hospital Medical Center, Cincinnati, OH 45229, USA; michelle.recto@cchmc.org; 4 Department of Pediatrics, University of Cincinnati, Cincinnati, OH 45229, USA

**Keywords:** anorexia nervosa, atypical anorexia nervosa, adolescents, eating disorders, obesity, weight suppression, youth

## Abstract

Atypical anorexia nervosa (AAN) is a restrictive eating disorder (ED) that describes individuals who may be normal weighted or overweight; many have a premorbid history of obesity. Pediatric care providers are trained to identify and provide best practices for youth with pediatric obesity; however, most pediatric care providers are not trained to assess and treat restrictive EDs which typically present in youth aged 10 and 14 years. Although individuals with AAN may appear to be within a ‘healthy weight’, many experience malnutrition, psychological symptoms, and severe physiological complications after weight loss. These individuals are presenting to pediatric services at an increasing rate and exhibit acute medical instability along with severe ED psychopathology. One complicating factor is youth with AAN may take longer to be identified by pediatric providers and may be reluctant to engage in treatment. Delayed treatment for AAN, along with all EDs often results in poorer treatment outcomes. A greater understanding of this complex illness is essential to inform medical decisions, such as labs, vitals, hospital admissions, and psychological therapy. Currently, there are no standardized guidelines for treating AAN in youths. This review is designed to present evidence-based treatment to inform and guide best treatment practices.

## 1. Introduction

Atypical anorexia nervosa (AAN) was added to the Diagnostic and Statistical Manual, fifth edition (DSM-5) under the category of Other Specified Feeding and Eating Disorder (OSFED) [1]. Those with AAN have all the features of Anorexia Nervosa (AN) such as restricting energy intake, intense fear of gaining weight, disturbance in one’s experience of body weight or shape, and a persistent lack of recognition of the seriousness of one’s current body weight [1]. What distinguishes AAN from AN is that despite meeting all the criteria for AN and losing a significant amount of weight, the individual’s weight is within or above the ‘normal’ weight range. However, like individuals with AN, those with AAN present with weight loss and may be severely malnourished with medical and psychiatric instability. This presents a challenge for health care providers to recognize and treat AAN appropriately as most providers have been trained to rely on low weight as a primary indicator of malnourishment and diagnosis of AN.

It is estimated that adolescents with a history of overweight or obesity represent approximately 36% of patients presenting for treatment of serious restrictive eating disorders (EDs) [2]. Males may be especially vulnerable as premorbid obesity is a risk factor for ED development in males [3]. One study found that youth with AAN who were previously overweight or obese experienced an increased rate and greater percentage of weight loss when compared to premorbid normal weighted adolescents with AN [4]. In addition, over the last ten years, clinicians have documented an increase of youth with AAN admitted to inpatient units [4,5,6]. These patients exhibited the same behaviors and cognitions as those with AN and similarly experienced acute medical complications. Medical instability appears to be just as severe if not worse in patients with AAN [5,6,7,8], and in one study those with AAN experienced more severe disordered eating behaviors and higher body image distress when compared to those with AN [5].

These youths with a higher BMI face specific challenges—individuals may initially receive positive reinforcement for their weight loss from friends, family, and healthcare professionals. Concerningly, health care providers may view the weight loss in these youth as a positive health outcome and fail to screen for the possible onset of a serious ED such as AAN. Once symptomatic, their distress also may not be viewed as seriously as low weighted individuals. People with less ‘stereotypical’ ED presentations are less likely to seek treatment and their symptoms are often unrecognized by primary care professionals [9,10]. A systematic review found that in clinical settings, individuals with AAN are undertreated compared to individuals with AN who received more referrals and treatment [11]. One study found that youth with EDs with a history in the overweight range took approximately 10 months longer to be diagnosed [2,12]. As a result, these individuals may be missed early in their illness and often present to care later with more significant weight loss [2,12]. Another study noted that 12% of patients with AAN who were pre-morbidly overweight or obese could have been diagnosed sooner [5]. This is concerning as youths who have higher body weights with subthreshold EDs may experience delayed diagnosis and treatment and could progress to full threshold AN. It is essential for health care providers to accurately assess weight loss in patients of any size to screen for ED symptoms and cognitions as delayed treatment results in poorer outcomes [5].

## 2. Methods

A comprehensive search of peer-reviewed journals was conducted by using key terms including “atypical anorexia”, “restrictive eating disorder”, “anorexia nervosa, adolescents”, “eating disorder not otherwise specified”, “subthreshold eating disorder”, “premorbid overweight and obesity”, “weight bias”, “and weight suppression”, in order to address the specific characteristics and treatment of AAN. Databases searched included, but were not limited to, PubMed and Google Scholar. The search was restricted to the titles and abstracts of English-language articles. Both adolescent and adult population articles were considered as data are limited regarding AAN in adolescents. Sixty-seven articles from 2007 to 2022 were selected based on the scope of this article which is focused on medical and psychological treatment related to AAN. Twelve additional articles were selected regarding ED assessment tools and psychopharmacological treatment ranging from 1982 to 2020.

## 3. Epidemiology

### Demographics and Prevalence

There is an overall lack of information regarding the prevalence of EDs in adolescents [13]. Although the diagnostic category of OSFED represents the largest proportion of ED diagnoses including AAN, purging disorder, night eating syndrome, and subclinical bulimia nervosa, it has received much less research attention compared to other EDs since its introduction in DSM-5 in 2013 [14,15,16,17]. One longitudinal study of Australian youths showed that when the DSM–IV–TR criteria were changed to DSM-5 criteria, AAN was identified in 0.3% and 0.9% of boys and girls aged 14 [18]. In this sample, OSFED diagnosis was greater at age 14 (approximately 50% in males and 40% in females) than at ages 17 or 20 (15% to 30%). Additionally, most EDs were associated with increased odds of being at a higher weight and were experienced across age, weight, socioeconomic and migrant status [18]. Another study of adolescents using DSM-5 criteria found that 3.6% of the participants met the criteria for AAN [19]. One multi-site study of 14 adolescent medicine clinics found that 33.9% of patients met the criteria for AAN while 53.6% met the criteria for AN [20]. A systematic review of AAN found that although prevalence rates varied across studies, epidemiological data showed a large portion of those who had ED diagnoses met the criteria for AAN [11]. It is hypothesized that the lack of consensus regarding positive treatment outcomes such as weight goals may be explained by differences in criteria for AAN from researchers and clinical practitioners [20]. Additionally, many have noted that the DSM-5 definition of AN vs. AAN is contradictory and confusing [11].

To address the increasing prevalence of AAN among youth with restrictive EDs, the American Academy of Pediatrics, American Society for Parenteral and Enteral Nutrition, Academy of Nutrition and Dietetics and the Society of Adolescent Health and Medicine recommended including percentage of weight loss in the assessment of malnutrition [21,22,23]. This is consistent with how the magnitude of body weight loss or weight suppression is used in the adult ED literature as an important indicator of the severity of illness in patients with AN [13] and has proven to be a useful tool for assessing disease severity and treatment outcome in youth with restrictive EDs [6].

It is important to note that the COVID-19 pandemic and subsequent public health measures have resulted in a worsening of ED symptoms and an increase in psychological distress [24]. It is hard to measure the future impact of the pandemic on youth; however, data has consistently shown increased and worsening ED symptomatology since the onset of the pandemic which may represent greater incidence, prevalence, and/or acuity of EDs during the COVID-19 pandemic [25,26,27]. Finally, a recent study found a 60% increase of new onset AN or AAN diagnoses in youth compared to pre-pandemic rates, with the incidence of AN/AAN increasing from 24.5 to 40.6 cases per month [28]. This has direct consequences for health care providers who may also be experiencing an increased demand for services and highlights the need for pediatric care providers to recognize and treat AAN quickly and effectively.

## 4. Medical and Psychiatric Sequela

### 4.1. Medical Complications

Medical complications in AAN mirror those with AN, as the primary pathophysiology of malnutrition remains the same: inadequate caloric intake to meet energy expenditure needs. Those with AAN appear to present with the same medical severity as those with AN. A study of 256 patients with EDs compared 118 with AN and 42 with AAN and found no difference in rates of bradycardia, orthostatic changes, hypothermia, nor hospital admissions [5]. The most concerning vital sign abnormalities that lead to hospitalization are sinus bradycardia and orthostatic hypotension, a manifestation of severe malnutrition resulting in increased vagal tone and decreased oncotic pressure. Patients can even experience myocardial atrophy; thus, care must be taken to monitor for fluid overload in these patients, which can precipitate congestive heart failure [29]. Following the same guidelines for youth with AN, hospitalization should be considered for youth with AAN when resting heart rate becomes less than 50 or blood pressure less than 80/50 mmHg [23].

Other medical complications that may require urgent medical intervention include electrolyte imbalances, particularly in patients with AAN, and concomitant purging from vomiting or laxative abuse. Hypokalemia in particular from purging behaviors puts these patients at significant risk of developing cardiac arrhythmias which can be fatal [29]. Laxative abuse can also cause electrolyte abnormalities such as hypokalemia or hypermagnesemia, which are associated with cardiac, renal, and nervous system injury [30]. Although electrolytes do not necessarily need monitoring at every encounter, monitoring at the first visit, when the frequency of purging changes, or with any clinical concerns are indicated. Table 1 lists some indicators of medical severity and possible hospitalization.

Outside of the most common urgent medical complications noted above, youth with AAN can experience medical problems in every organ system. Most commonly, providers will find gastrointestinal distress, reversible laboratory findings consistent with malnutrition, and amenorrhea [31,32,33]. Frequent complaints of gastrointestinal distress may be secondary to constipation, organic causes are often secondary to dysmotility from malnutrition, or functional abdominal pain [29,32,34]. However, these symptoms can be confusing, as the abdominal pain often has no clear medical cause and will improve as patients progress through their treatment [29,32]. Malnutrition can also manifest as bone marrow suppression, causing a reduction in all cell lines including anemia, thrombocytopenia, and leukopenia. This is suspected to be due to bone marrow atrophy but is fully reversible with re-nutrition [35]. Thyroid labs may also be abnormal, with findings consistent with sick-euthyroid. It is fully reversible with weight gain and are a result of the body’s attempt to reduce metabolic needs and conserve energy [36]. Regardless, abnormal labs should be re-evaluated and not solely attributed to AN or AAN if weight gains are not consistent with reported nutritional intake—in order to not miss a pathological problem such as malignancy or thyroid disease. 

Lastly, malnutrition can also lead to hypothalamic suppression causing hypogonadotropic hypogonadism and secondary amenorrhea, although this finding is less common in AAN than in AN [5,29]. For the large majority (85%) of females with AN, ovulation and fertility returned with weight recovery. Of note, those who had persistent amenorrhea were practicing significantly more intense physical activity than those whose periods returned [33]. Furthermore, as a result of estrogen deficiencies, patients with AAN are also prone to developing osteopenia and osteoporosis leading to an increased risk of pathologic fractures [29]. 

### 4.2. Psychiatric Complications

Patients with AAN appear to have similar ED thoughts and behaviors as patients with AN and can experience similarly severe psychiatric symptoms [5,29,37,38]. One study found that patients with AAN had ED cognitions (i.e., fear of gaining weight, focus on shape and weight, preoccupation with food and eating) at the same level as patients with AN measured by the Eating Disorder Examination—Questionnaire (EDE-Q) [37]. Another study of adolescents treated at a specialty pediatric program found that those with AAN reported more severe ED cognitions and behaviors than those with AN on all subscales of the EDE-Q youth version [5]. The most common psychiatric comorbidities in this cohort were depressive disorders, anxiety disorders, and obsessive-compulsive disorder [5]. Of the AAN patients, 38% had a psychiatric comorbidity and 43% endorsed self-harm and suicidal ideation [5].

Of note, premorbid overweight and obesity is an important factor to consider regardless of the diagnoses of AN vs AAN for youths. A recent study showed that adolescents with AN/AAN with premorbid overweight or obesity endorsed greater ED severity and scored higher on the EDE-Q Global scale, the EDE-Q Restraint, Weight Concerns, and Shape Concerns subscales than premorbid normal weighted AN patients [38]. Those with AAN also experienced greater psychological morbidity on scales for anxiety and depression [38]. Given the psychiatric comorbidity as well as the significant impact of malnutrition on health, all individuals who have lost weight must be screened for ED symptoms regardless of body weight and size, or lack of previous psychiatric history [7].

## 5. Assessment

### 5.1. Medical Assessment and Treatment Recommendations

Diagnosis and evaluation of AAN can be complicated by the lack of clear delineation in weight criteria between AN and AAN. Previous iterations of the definition for AN included an 85% ideal body weight (IBW) cutoff, although they did not define how IBW would be defined (e.g., BMI, growth chart, pre-morbid weight). The IBW cutoff was removed from the most recent DSM definition of AN to give clinicians more leeway in determining unhealthy patterns of weight loss [1]; however, there are consequently no concrete guidelines to separate “significant low body weight” from the normal or above normal weight range for AAN [29]. Evidence suggests that even a 5% weight loss in the setting of cognitive dysfunction can be associated with a clinically significant eating disorder, and some experts have argued for doing away with weight criteria altogether [39,40]. It is important to obtain a thorough weight history, which can serve as a good predictor of illness severity in both AN and AAN. A study of weight history and illness severity in adolescents and young adults with AN and AAN demonstrated that a greater amount, rate, and duration of weight loss were all associated with worse medical and nutritional status, challenging the dogma that low BMI alone was the only or most significant predictor of outcomes [41].

Intake of all patients with AAN should involve a full baseline history and physical including assessment of vital signs (including orthostatic vitals) and baseline height, weight, and BMI. The physical exam is an opportune time to evaluate for signs of purging and other disordered behavior. Organic causes of weight loss should be ruled out in the work of AAN, including endocrinologic disease, chronic illnesses, malignancy, inflammatory bowel disease, and celiac disease [23]. All patients should get a full laboratory evaluation as well to assess blood counts, electrolyte abnormalities, liver function tests, and thyroid function tests as well as markers of nutritional status including albumin, prealbumin, and transferrin. Certain patients may warrant hormone testing and pregnancy testing if amenorrhea is present. All patients should have an EKG performed as well to rule out cardiac dysrhythmias; specific patients with severe malnutrition may warrant an echocardiogram if cardiomyopathy or pericardial effusion is suspected. 

### 5.2. Weight Restoration and Pharmacological Treatment

The medical treatment of AAN, as in any restrictive ED, is to increase caloric consumption in order to reverse malnutrition. As a result, weight gain is as critical an aspect of treatment for AAN as it is for AN, even for patients who are above the normal weight range [29]. Care must be taken in the initial stage of treatment to avoid the risk of refeeding syndrome; as such, a lower daily caloric goal of 1000–1400 kcals per day is often recommended as a starting point and can be increased by up to 400 kcals every 3–4 days as appropriate [29,42]. Significant exercise and physical activity must also be discontinued until an appropriate amount of weight gain has been achieved [43]. Notably, the standard of practice has been shifting towards starting at a higher caloric intake of 1750–2000 kcals per day, with or without prophylactic phosphorus supplementation [44]. This is because studies are showing that higher-calorie treatment plans are not associated with an increased risk of developing refeeding syndrome or readmission to the hospital and even lead to faster stabilization of vital signs and shorter hospital stays [45,46]. The expected achievable rate of weight gain varies by treatment disposition; about a pound per week with weekly outpatient treatment, two pounds per week in day treatment programs, and three pounds per week is possible in an inpatient setting [29]. 

Determining a goal weight can be difficult, especially for those with AAN. For pre-pubertal children, the overarching goal is to return patients to an appropriate trend on their growth curve. For adolescents, the goal weight can be determined by calculating the weight for a BMI at the 50th percentile. For AAN patients whose weight is above that mark, a midpoint between the 50th percentile and their maximum weight can be determined as their goal weight [29]. It is important to re-evaluate goal weights throughout the course of treatment while evaluating overall clinical progress and nutritional status.

In terms of pharmacologic management of AAN, there is a paltry amount of evidence supporting or refuting the use of medication. Selective serotonin uptake inhibitors (SSRIs) have been shown to have limited use in the treatment of AN for either weight gain or management of depression or anxiety, although, in patients with bulimia nervosa (BN), evidence suggests SSRIs can help alleviate depressive symptoms [29,47,48]. As such, patients with AAN who engage in binging or purging behaviors or have a more significant component of anxiety or depression may appropriately be trialed on an SSRI. Atypical antipsychotics, such as olanzapine, have been used in refractory and severe AN has shown some benefit; however, it may not be appropriate to translate this body of literature to AAN as these patients are at risk for developing significant rebound weight gain [29].

### 5.3. Psychiatric Assessment

#### 5.3.1. General Assessment

It is best to conduct a comprehensive psychiatric assessment that includes youth and their caregivers. The general psychiatric assessment should include a full history of present illness and attempt to identify any precipitating factors and timeline of symptoms. Clinicians also need to gather detailed past psychiatric history, including current and past diagnoses, premorbid and current OCD traits/behaviors, trauma history, and substance use (e.g., alcohol, vaping, drug use, stimulants), as well as any previous psychiatric treatment (e.g., level of care, psychiatric medications). The assessment also needs to include information related to developmental (e.g., developmental milestones), social (e.g., bullying, peer relationships, impact of COVID-19), family (e.g., family psychiatric history, family relationships), and academic history (e.g., IEP/504 plan, sports, student clubs). In addition, it is important to assess current as well as past safety issues including non-suicidal self-injury (NSSI) and suicidality including ideation, intent, attempts, and access to means. Patients with AN have high mortality rates and suicide is one of the main causes of death [49]. Lastly, a full mental status exam, including the patient’s level of judgment, insight, and any cognitive effects of malnutrition, should be assessed. 

When assessing ED thoughts and behaviors, it is important to be concrete and specific. Most patients will express fear of gaining weight and body image distortion. Evaluation of body dissatisfaction and preoccupation in males should focus on their beliefs about muscularity and leanness rather than relying on a standardized tool which may not capture full diagnosis nor their psychological distress as traditional assessment tools measure symptoms that are more specific to females [50]. Additionally, younger patients may have difficulty elucidating their cognitions related to the function of their ED behaviors—it is essential to involve parents in these discussions. Younger patients may not meet the full criteria for a DSM 5 diagnosis, nor endorse ED cognitions, however, as noted, it is still important for these youths to restore their growth curves [43]. Please see Table 2 for suggestions for the ED assessment.

#### 5.3.2. Validated Measures

There are several validated measures that healthcare providers can use to diagnose EDs. Although it is best to first conduct a comprehensive assessment as described above, some healthcare providers may not have time to score longer validated measures and may choose to use brief screeners. A positive screening on either of the brief screeners should be followed by a comprehensive medical and psychological assessment [34,51]. Options are listed below.

##### Brief Screeners

- The five-item SCOFF (Sick, Control, One, Fat and Food) questionnaire is a widely administered screening measure used by primary care physicians [34,52]. However, a recent meta-analysis concluded that while the SCOFF is an effective tool for identifying some EDs (i.e., AN and BN) in young women with ED symptoms, there is not enough evidence to support administering the SCOFF to screen for full DSM 5 ED diagnoses in primary care and diverse community-based settings [52].
- The Eating Disorder Screen for Primary Care (ESP) is a brief four-question screening assessment used to identify primary care patients who are at risk for EDs and in need of specialized care [51].

##### Self-Report Questionnaires

- The Eating Disorder Examination-Questionnaire youth version (EDE-Q) assesses ED symptoms from the past 28 days and is a self-report version of the Eating Disorder Exam (EDE). The EDE-Q has 28 items and is quicker to administer than the EDE. The EDE-Q has good psychometric properties with internal consistency reported to be high (α > 0.90) in children over 12 and adolescents however, the EDE-Q has not been validated for children under 12 [53,54,55].
- The Child Eating Disorder Examination (ChEDE)—is a tool that has been adapted from the EDE for use in youth aged 8 to 14 years [56,57]. The ChEDE has 22 items, is a self-report measure and its psychometric properties have been confirmed.
- The Eating Attitudes Test (EAT-26) is a statistically valid measure of 26 items used to evaluate the cognitive, emotional, and behavioral patterns of eating [58]. The higher the result, the more serious the ED cognitions and behavior problems. A total score of 20 or more may indicate an ED.
- The Eating Disorders Inventory 3 (EDI-3) used for ages 13 to adult is a widely used self-report measure shown to be clinically relevant in individuals with EDs [59]. The EDI-3 has 91 items and measures the psychological symptoms associated with EDs.
- The Development and Wellbeing Assessment (DSWBA) is designed to generate ICD-10 and DSM psychiatric diagnoses for 5–16-year-olds. The DSWBA uses both parent and patient self-report and may provide information about possible psychiatric comorbidities [34,60].

### 5.4. Therapeutic Approaches

There are limited data evaluating treatment approaches for youth with EDs and even less information for youth with AAN. Currently, family-based treatment (FBT) is the first-line approach for youth with restrictive EDs [23,61]. Since patients with AAN share more similarities than differences with patients with AN, researchers have suggested that FBT may be a helpful intervention for patients with AAN [29]. 

FBT is a structured time-limited manualized outpatient therapy for EDs designed to empower caregivers to restore their child to full health. The first tasks of therapy include weight restoration and a reversal of ED behaviors and cognitions [62]. Caregivers are put in charge of all meals with treatment consisting of three phases to achieve these goals: weight restoration, stabilization, and maintenance [62]. A recent qualitative study with mental health practitioners found that for patients with AAN, lack of clarity around the term ‘significant weight loss‘ and weight recovery presented challenges [63]. The authors concluded that further research would be helpful to assess degrees of malnutrition to inform adaptions of FBT for patients with AAN [63]. Indeed, an earlier study found that patients with AAN treated with FBT experienced a significant decrease in ED symptoms with no significant change in percent of median BMI for age and gender [64]. While the data demonstrated the effectiveness of FBT in addressing some ED symptomatology, the data is not clear regarding the percentage of weight recovery needed for patients with AAN [63,64]. This lack of consensus among clinicians and families complicates the goals of treatment further demonstrating that research is needed to define and assess ‘good outcomes’ for psychological treatment for patients with AAN. However, FBT is still the treatment of choice for most patients with restrictive EDs who need to restore weight [23,61]. 

Adolescent-focused therapy (AFT) has been shown to be an effective treatment for youth with AN [65,66]. This treatment helps the youth identify and cope with negative emotions and developmental challenges that may contribute to the ED [65].

Cognitive behavior therapy—Enhanced (CBT-E) has been proposed as an alternative treatment for youth with AN when FBT is not feasible [67,68]. CBT-E actively involves patients to achieve a healthy weight and target the psychopathology that underlies their ED [68]. Parents are involved in the initial stages of the treatment and their role is to support their child in individual treatment [68]. A recent study that evaluated CBT-E in a clinical setting found that it was a suitable treatment for youth with AN and resulted in positive outcomes [69].

Dialectical behavior therapy (DBT) incorporates CBT and mindfulness-based skills to increase interpersonal effectiveness, develop emotion regulation, and build distress tolerance skills [70]. DBT shows effectiveness for patients with binge eating disorder (BED) [71], however, less is known about the efficacy of DBT for youth with AAN. One study incorporated DBT strategies with FBT for youth with AN [72] and demonstrated positive treatment outcomes [72]. Patients had significant increases in weight and had some improvements in ED psychopathology [72]. However, this was a pilot study, and more research needs to be conducted to further evaluate DBT as a treatment for youth with AN and AAN.

Overall, it is recommended that an early, intensive, and family-based approach appears to have the best overall outcomes for youth with restrictive EDs [23,55,61]. In addition to these treatment approaches that can be applied in the outpatient setting, providers may also consider higher levels of care, including intensive outpatient, residential treatment, and ED inpatient depending on the acuity of the ED symptoms, psychiatric co-morbidity, and medical instability.

## 6. Discussion

When treating youth with ED and premorbid overweight/obesity, it is important to consider weight-based stress, bias, and stigma unique to this population as these are present in the general public as well as among healthcare providers [73,74]. For example, those with AAN reported more weight-based teasing by family members and/or their peers [75]. They may also be vulnerable to anti-obesity messaging (e.g., such as from educational curriculum, media/internet, health care providers, family, and peer bullying as it was identified as one of contributing factors to the onset of EDs [76]. Patient’s history of overweight/obesity can also make it challenging for providers to help families understand that their child could still have a serious ED without looking emaciated [75], and balance the youth’s trauma of weight-based teasing with the urgent need for weight restoration. It can be particularly challenging to get treatment buy-in from both the youth and the family [75].

Unfortunately, there is a dearth of information regarding best practices for health care practitioners when discussing weight with their patients. However, the American Academy of Pediatrics (AAP) in response to weight stigma within healthcare settings has recommended using sensitive and non-stigmatizing language when counseling about weight [77]. The AAP recommends using neutral and person-centered language which places the youth before the diagnosis (an individual with an ED) [77].

One recommended approach that may be helpful with youths is a weight-neutral stance [77,78]. A study found that youth prefer non-stigmatizing language (e.g., avoid terms such as fat, chubby, skinny, anorexic) and a focus on health and behavioral solutions rather than weight [79]. Weight-related conversations should be non-shaming, and weight should not be associated with morality, appearance, or comparisons with others [79]. The study researchers concluded that patient-centered approaches that allow youth to discuss their own internal motivation, goals, and health concerns could be helpful for behavior change [79] which is essential for recovery from an ED. More research focused on using a health inclusive family-centered approach for assessment, treatment goals, and weight restoration for youth with AAN is needed. Additionally, researchers recommend that future samples should include diverse clinical populations such as gender-diverse individuals, individuals of color, and nonconforming individuals to fully understand the variations or lack of in AAN presentation [11,13].

## 7. Conclusions

It is important to correct the misperception that AAN is less serious than AN, as noted there are more similarities than differences between both diagnoses. Pediatric health care providers are often the first line providers to come across ED thoughts and behaviors in youth and are an essential part of the treatment team and their patient’s recovery. It is important to note that rapid and total weight loss and malnourishment in youth, not appearance, is indicative of the seriousness of the illness and life-threatening complications can be present at any weight. Further research is needed to address the classification of AAN so that concrete medical guidelines and psychiatric interventions can be developed and operationalized. It is challenging for researchers to clearly understand the true prevalence and incidence of AAN when there are varying approaches to classifying AAN based on weight criteria rather than total weight loss, weight suppression, and ED cognitions. Additionally, quite often these youths have been caught between the fields of pediatric obesity and EDs and initially may have had ‘good intentions’ when first losing weight. A greater understanding of how EDs develop in the context of obesity will help guide health care professionals to intervene early and effectively which will improve treatment outcomes. While it is challenging to restore weight for those with AAN due to societal pressures and a lack of specific guidelines, viewing each patient as unique and deserving of treatment will shift all to a weight-neutral approach for treatment and recovery.

## Figures and Tables

**Table 1:** Indications for urgent evaluation and hospitalization.

**Malnutrition and Weight Loss**
- <75% of previous body weight
- Loss of >10% of body weight in <6 months
- Refusal to eat or drink in over 24 h
**Vital Sign Abnormalities**
- Heart rate < 50 beats per minute during the daytime
- Systolic blood pressure < 90 mmHg
- Orthostatic changes (with symptoms of pre-syncope) and pulse increase > 20 beats from lying to standing or systolic blood pressure drop of >10 mmHg
**Clinical Findings**
- Recent syncope
- Chest pain
- Seizure
- Dehydration
- Intractable vomiting
- Bloody emesis or diarrhea
- Severe (especially acute onset) abdominal pain
**Laboratory Abnormalities**
- Abnormal EKG (e.g., T wave abnormalities, QTc prolongation)
- Electrolyte derangements (e.g., hypokalemia, hyponatremia, hypophosphatemia)
- Severe hypoglycemia

Note. Adapted from AAP and SAHM guidelines.

**Table 2:** Eating Disorder Assessment.

Domains	Questions
**Restrictive Eating**	Highest, lowest, and current weight
	Restriction of energy intake: Are you dieting? What is the family culture regarding food and eating? What food groups will you not eat? Vegetarian or vegan?
	Calorie counting, weighing: Do you have a goal of daily calories? Do you weigh your food? Do you track your food/calories? Do you read labels?
	Fear of gaining weight?
	Desired weight: What is your goal weight?
**Compensatory behaviors**	Purging? If so what type? Vomiting, laxatives, enemas, insulin, or excessive exercise. Specify methods, frequency, and amount.
**Binge Eating**	Have you ever binged? Did you feel a loss of control when eating? Did you eat an objectively large amount in a short period of time? Do you eat alone and/or until uncomfortably full? Do you feel guilty after eating? Did you feel out of control when eating? Query for specific foods and amount.
**Avoidant/Restrictive Eating**	Probe if food restriction is based on appearance, texture, lack of interest, or aversive experience. If yes, query if there is marked social functioning, reliance on supplements and a history of failure to thrive.
**Body Image and Body Dysphoria**	Ask about perception of body, fear of gaining weight, perceived flaws, body parts that should be changed, body comparison to others, body preoccupation, body checking, and frequent weighing.
**Body Image specific to male-identifying youths**	Ask about the desire for thinness, leanness, and muscularity. Do you use appearance enhancing or performance-enhancing drugs or supplements, i.e., protein drinks?
**Other Eating Behaviors**	Physical symptoms (e.g., functional abdominal pain or vomiting) Food allergies or food preferences Relationship with food, cooking, rituals Excessive caffeine intake Excessive use of social media Chewing and spitting Rumination Hiding Food Body checking
**24-h Diet Recall**	Specify amounts and brands.

## Data Availability

Not applicable.
